# Temperature-Responsive Hydrogel for Silver Sulfadiazine Drug Delivery: Optimized Design and In Vitro/In Vivo Evaluation

**DOI:** 10.3390/gels9040329

**Published:** 2023-04-13

**Authors:** Maha Mohammad AL-Rajabi, Yeit Haan Teow

**Affiliations:** 1 Faculty of Chemical Engineering Technology, Universiti Malaysia Perlis, Arau 02600, Perlis, Malaysia; 2 Centre of Excellence for Biomass Utilization, Universiti Malaysia Perlis, Arau 02600, Perlis, Malaysia; 3 Department of Chemical and Process Engineering, Faculty of Engineering and Built Environment, Universiti Kebangsaan Malaysia, Bangi 43600, Selangor Darul Ehsan, Malaysia; 4 Research Centre for Sustainable Process Technology (CESPRO), Faculty of Engineering and Built Environment, Universiti Kebangsaan Malaysia, Bangi 43600, Selangor Darul Ehsan, Malaysia

**Keywords:** biocellulose hydrogel, temperature-responsive, drug delivery, response surface methodology (RSM), in vitro/in vivo performance

## Abstract

Response surface methodology (RSM) was applied to optimise a temperature-responsive hydrogel formulation synthesised via the direct incorporation of biocellulose, which was extracted from oil palm empty fruit bunches (OPEFB) using the PF127 method. The optimised temperature-responsive hydrogel formulation was found to contain 3.000 *w*/*v*% biocellulose percentage and 19.047 *w*/*v*% PF127 percentage. The optimised temperature-responsive hydrogel provided excellent LCST near to the human body surface temperature, with high mechanical strength, drug release duration, and inhibition zone diameter against *Staphylococcus aureus.* Moreover, in vitro cytotoxicity testing against human epidermal keratinocyte (HaCaT) cells was conducted to evaluate the toxicity of the optimised formula. It was found that silver sulfadiazine (SSD)-loaded temperature-responsive hydrogel can be used as a safe replacement for the commercial SSD cream with no toxic effect on HaCaT cells. Last, but not least, in vivo (animal) dermal testing—both dermal sensitization and animal irritation—were conducted to evaluate the safety and biocompatibility of the optimised formula. No sensitization effects were detected on the skin applied with SSD-loaded temperature-responsive hydrogel indicating no irritant response for topical application. Therefore, the temperature-responsive hydrogel produced from OPEFB is ready for the next stage of commercialisation.

## 1. Introduction

Hydrogels have attracted attention over the last 50 years as an effective and easy-to-apply drug delivery system owing to their favorable properties of absorbing large amounts of water [1]. The hydrogel polymeric structure can be modified to obtain the desired functionality, such as spontaneous response to temperature [2,3]. Temperature-responsive hydrogel is useful as a dermal drug delivery system because it shows a transition in its phase at a particular temperature, causing a sudden change in solubility [4]. Pluronic F127 (PF127) is a temperature-responsive polymer with potential use in the synthesis of temperature-responsive hydrogels. PF127 polymer is in sol-phase below the phase-transition temperature and changes to gel-phase upon increasing the temperature above the phase-transition temperature [5]. Owing to this phase transition characteristic, the formulation of temperature-responsive hydrogel could be designed such that the temperature-responsive hydrogel is in sol-phase at room temperature to fill the wound surface and transforms into a rigid hydrogel at the temperature of the human body. The rigid temperature-responsive hydrogel at human body temperature acts like a solid artificial barrier with a sustained release feature. Moreover, PF127 as a wound dressing could be used as a cleaner to remove dead tissues and/or debris that are generated from the wound [6]. PF127 also exhibits surfactant properties—this can help in improving the interactions with hydrophobic drugs and cell membranes owing to PF127′s hydrophobic and hydrophilic domains; thus, it plays a vital role in drug delivery systems [7,8]. However, despite the multiple benefits of PF127, it cannot stand alone for hydrogel formation because of its poor mechanical properties [9] To circumvent these problems, other polymers (synthetic or natural) have to be added into the hydrogel network to enhance its mechanical strength.

Unfortunately, petroleum-based and synthetic cellulose-added polymers used in hydrogel synthesis are expensive, highly toxic, and non-biodegradable [10,11]. The hydrogels from these base materials can create environmental problems related to waste disposal after usage [12]. Therefore, with the purpose of protecting the environment, as well as reducing environmental impacts, additional efforts should be made to find a sustainable means of developing a natural and environmental friendly hydrogel to substitute for current synthetic polymer-based hydrogels for different applications, such as drug delivery systems [13]. Recent advances in the utilisation of biocellulose extracted from agricultural wastes has greatly expanded the avenues for hydrogel synthesis. However, the synthesis processes for biocellulose hydrogels involve the use of toxic cross-linkers; unreacted toxic cross-linkers retained in the hydrogel matrix may cause skin irritation and sensitisation during application. In addition, although biocellulose hydrogel is easier to administer compared to conventional drug delivery systems, its flat stagnant sheet structure is not able to cover uneven wound/burn surfaces completely, creating risk of site infection. Furthermore, stagnant sheet biocellulose hydrogels have the drawbacks of a complicated drug loading process, low holding, and difficult drug release control kinetics for application as a drug delivery system. Therefore, the synthesis of functionalised biocellulose hydrogels using a green chemical process, which can easily flow at body temperature, with sustained release properties with respect to the temperature stimulus, are required for better drug delivery system development.

It has been estimated that 75% of deaths in patients with burns are due to infection [14]. Every year, more than 180,000 people die due to burn wounds in low- to middle-income countries [15]. Although many therapeutic treatments have been introduced for wound healing with significant advances in wound care and treatment, incidence and death rates have increased, due to a large extent to ensuing microbial infections in patients with burns. Silver sulfadiazine (SSD), combining sulfadiazine with silver, is widely employed as an antibacterial agent for burn management [16]. The antimicrobial activity of SSD develops by the degradation of SSD molecules into sulfadiazine and silver ions. The silver ion of the SSD interrupts the triphosphate synthesis in the bacteria, whereas the sulfadiazine inhibits the synthesis of folic acid in the bacteria. Folic acid plays an essential role in the growth and reproduction of bacteria [17]. Once the bacteria’s synthesis of folic acid is inhibited by sulfadiazine, the DNA replication of the bacteria is also inhibited, which results in interference with the replication of the bacteria, followed by bacteria cell death.

Generally, the temperature-responsive hydrogel′s properties and performance are influenced by the concentration of polymers in its matrix [18]. The most important factor for the sol-to-gel transition at the phase-transition temperature, also known as the lower critical solution temperature (LCST), is the temperature-responsive polymer concentration. At low PF127, the weight percent in the temperature-responsive hydrogel formulation will prevent micelle formation of PF127 unimers, so gelation will not occur [19]. On the other hand, a higher PF127 percent in the temperature-responsive hydrogel means more micelles are produced, which will lead to the aggregation of unimers of PF127, promoting the formation of a solid-like hydrogel at lower temperatures [20]. The LCST of temperature-responsive hydrogels is also affected by the percentage of biocellulose in the matrix. LCST decreases with increasing biocellulose weight percent [20]. Apart from LCST, temperature-responsive hydrogel composition affects its mechanical strength reflected in the storage modulus, the half-life of drug release (t_50%_), and the inhibition zone diameter against bacteria. However, there have been few studies on the optimization of temperature-responsive hydrogel formulation by manipulation of its formulation. This optimization is considered vital to engineering design and to ensure superior performance in drug release.

The purpose of the present work was to optimise the formulation of temperature-responsive hydrogels using a response surface methodology (RSM) and to test the toxicity and biocompatibility of the optimised formula. The optimum formulation was determined based on drug delivery performance. Independent process variables, including biocellulose percentage and PF127, were manipulated to optimise the lower critical solution temperature (LCST), storage modulus (G′), t50%, and the inhibition zone diameter against *Staphylococcus* of the temperature-responsive hydrogel. Furthermore, confirmatory experimental procedures were performed under optimal conditions for comparison with the predicted response. Moreover, to evaluate the toxicity of the optimum temperature-responsive hydrogel, in vitro cytotoxicity testing against human epidermal keratinocyte cells was performed. Finally, in vivo (animal) dermal testing, including dermal sensitization and animal irritation, was performed to evaluate safety and biocompatibility.

## 2. Results and Discussion

### 2.1. Optimisation of Temperature-Responsive Hydrogel Formulation Using Response Surface Methodology (RSM)

RSM was used to find the optimum formulation of a temperature-responsive hydrogel. Two independent process variables, namely, biocellulose percentage (0.0–3.0 *w*/*v*%) and PF127 percentage (15–35 *w*/*v*%), were studied. Four responses, namely, LCST, G′, t_50%_, and inhibition zone diameter against *S. aureus*, were selected to indicate the suitability of the temperature-responsive hydrogel as a drug delivery system.

**Figure 1:**
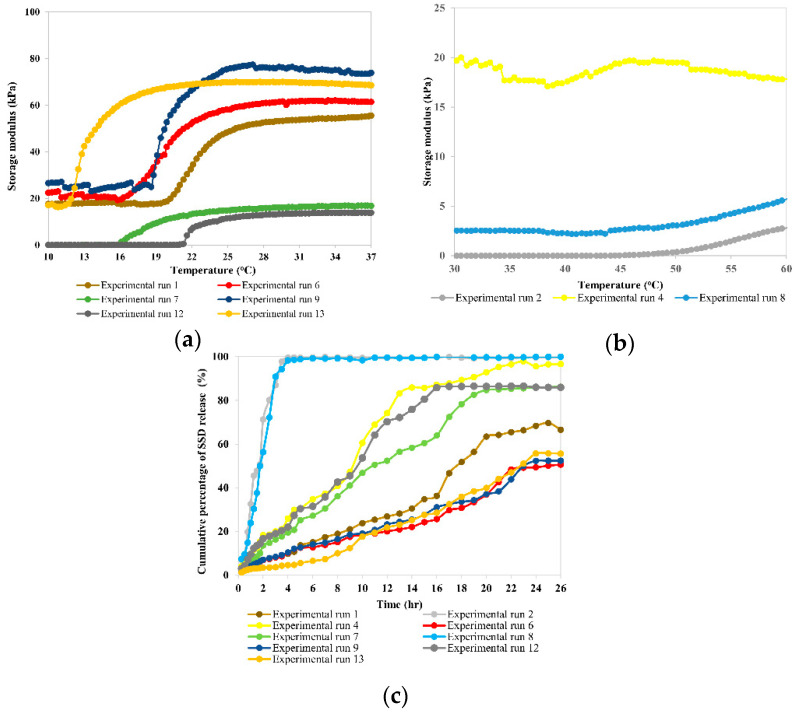
Storage modulus (G′) of temperature-responsive hydrogels at temperature (**a**) 10–37 °C and (**b**) 30–60 °C. (**c**) Cumulative percentage of SSD release from temperature-responsive hydrogels.

**Figure 2:**
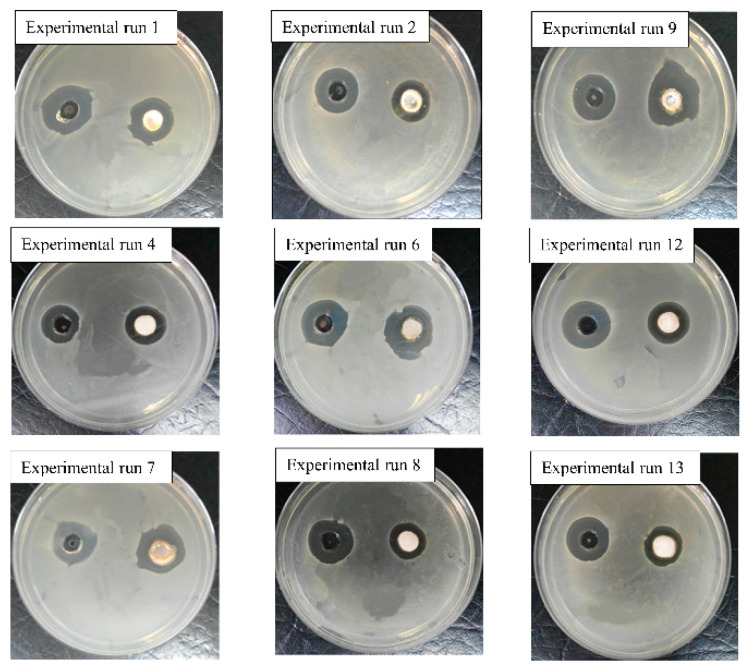
Photographs of inhibition zone on SSD-loaded temperature-responsive hydrogels against *S. aureus*.

The G′ of temperature-responsive hydrogels as a function of temperature, and the cumulative percentage of SSD release from temperature-responsive hydrogels, are illustrated in Figure 1. Figure 2 shows photographs of the inhibition zone on SSD-loaded temperature-responsive hydrogels against *S. aureus.* The CCD and responses for the temperature-responsive hydrogel formulations are summarised in Table 1. The LCST values recorded in Table 1 refer to the onset of a G′ increment as a function of temperature [21] shown in Figure 1. The LCST of experimental runs 2, 8, and 4, with PF127 percentage ≤15.00 *w*/*v*%, was not detected in the temperature range 10–37 °C, as shown in Figure 1a. Consequently, G′ was measured at an extended temperature range between 30–60 °C, as shown in Figure 1b. The G′ values recorded in Table 1 refer to the G′ obtained at 37 °C, while the t_50%_ values refer to the half-life of SSD release obtained from Figure 1c. As shown in Table 1, the LCST was attained in experiment run 8 at 1.50 *w*/*v*% biocellulose and 10.86 *w*/*v*% PF127. On the other hand, the lowest LCST was attained in experiment run 13 at 1.50 *w*/*v*% biocellulose and 39.14 *w*/*v*% PF127. PF127 has a higher impact on the LCST of the temperature-responsive hydrogel as PF127 is a temperature-responsive polymer. At a high percentage of PF127, more PF127 micelles in the same volume and hydrogen bonds occur, promoting the formation of a solid-like hydrogel at low temperature [22]. This explains the decrease in LCST with increase in the PF127 percentage at a constant biocellulose percentage.

The lowest G′, t_50%_, and inhibition zone diameter were obtained in experiment run 2 at 15.00 *w*/*v*% PF127 without biocellulose. The highest G′ and inhibition zone diameter were attained in experiment run 9 at 3.62 *w*/*v*% biocellulose and 25 *w*/*v*% PF127. However, the highest t_50%_ was attained in experiment run 6 at 3.00 *w*/*v*% biocellulose and 35.00 *w*/*v*% PF127. Both the biocellulose percentage and the PF127 percentage were inter-related in affecting the values of G′, t_50%_, and the inhibition zone diameter. In general, the G′ and t_50%_ of the temperature-responsive hydrogel were increased with increase in the biocellulose percentage in the hydrogel formulation. Biocellulose binds with hydrophilic poly(ethylene oxide) in PF127 chains through intermolecular hydrogen bonding [23]; this would increase the cross-linking density of temperature-responsive hydrogels, thus giving a higher G′ value. In addition, the high weight percentage of biocellulose in the temperature-responsive hydrogel’s formulation led to strong interlocking of SSD within the hydrogel network, prolonging the release of SSD. Conversely, the inhibition zone diameter of the temperature-responsive hydrogel against *S. aureus* was increased with increase in both biocellulose percentage and PF127 percentage in the hydrogel formulation. This could be explained by the sustained release of the SSD drug from the temperature-responsive hydrogel at high biocellulose and PF127 percentages. Sustained SSD drug release maintains a localized and constant drug presence; this results in improving the drug inhibitory potential against bacterial strains [24,25].

### 2.2. Statistical Model and Analysis of LCST

The results of ANOVA of a quadratic model for LCST are shown in Table 2. The Prob > F value of the quadratic model had a value <0.0001 (referred to Table 2), which was smaller than 0.05, indicating the significance of the developed quadratic model for LCST at the 95% confidence level [26]. Furthermore, the Prob > F value was 0.0915 (>0.05) for lack of fit, signifying that it was insignificant for the quadratic model that was developed. This implies that the developed quadratic model was appropriate for relating the independent process variables to the responses. The quadratic model consisted of five model terms, A, B, AB, A^2^, and B^2^. The ranking of these terms based on significance was arranged according to the Prob > F value in accordance with B and B^2^ > A > A^2^ > AB. When the value of Prob > F for a specific factor is smaller, this means that this factor is more significant [27]. As expected, the PF127 percentage (B) was the most significant model term in predicting the LCST of the temperature-responsive hydrogel due to the temperature-responsive properties of PF127.

The high correlation coefficient, R^2^ (0.9935) and adjusted R^2^, R^2^_adj_ (0.9889) of the developed quadratic model reflect its excellent validity and reliability. The predicted R^2^ with a value of 0.9611 was close to R^2^_adj_. In addition, the value of adequate precision indicated that the signal-to-noise ratio was 41.0773. This value is greater than 4, demonstrating that the developed model has an adequate signal for predicting LCST within the range of study [28]. Equation (1) shows the quadratic model for LCST in terms of actual factors after excluding the non-statistically significant model terms.

(1) $$Y_{1}=+32.98-3.24A-42.04B+1.58A^{2}+21.25B^{2}$$

subjected to: 0 *w*/*v*% ≤ A ≤ 3.62 *w*/*v*%, and 10.86 *w*/*v*% ≤ B ≤ 39.14 *w*/*v*%

**Figure 3:**
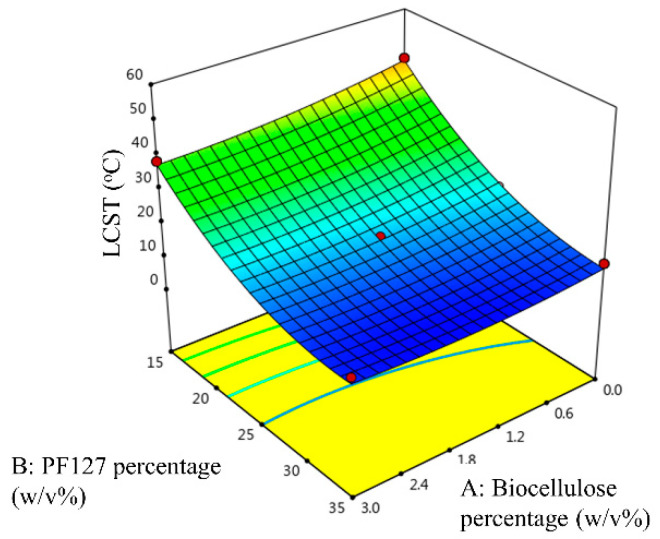
Three-dimensional response surface of LCST as a function of biocellulose percentage and PF127 percentage.

Figure 3 shows the three-dimensional response surface of LCST as a function of the biocellulose and PF127 percentage. As shown in Figure 3, high LCST was attained at a low biocellulose percentage and a low PF127 percentage. The LCST of the temperature-responsive hydrogels was decreased with increase in PF127 percentage, which is in good agreement with our earlier study [20]. An increased number of PF127 micelles contained in temperature-responsive hydrogel matrix will increase hydrogen bonding and promote the formation of a solid-like hydrogel at low temperature [22]. Furthermore, the LCST of the temperature-responsive hydrogel decreases with increase in the biocellulose percentage in the hydrogel formulation. This is because biocellulose binds with hydrophilic poly(ethylene oxide) chains through intermolecular hydrogen bonding [23]. The bonding between biocellulose and hydrophilic poly(ethylene oxide) chains promotes dehydration of these chains as it decreases the hydrogen bonding between poly(ethylene oxide)-water molecules. This causes an increase in entanglement of adjacent P127 micelles, leading to the formation of gelation at lower temperatures [23].

#### 2.2.1. Statistical Model and Analysis of G′

The ANOVA results for the quadratic model for G′ are shown in Table 3. The Prob > F value of the quadratic model was smaller than 0.05 (<0.0001 referred to Table 3) indicating the significance of the developed quadratic model for G′ at the 95% confidence level [26]. The Prob > F value for lack of fit was 0.0519 (>0.05). Regarding the model terms, the developed quadratic model contained five model terms, A, B, AB, A^2^, and B^2^. Based on the Prob > F value for each model term, the significance ranking was arranged in accordance with A and B > B^2^ > A^2^ > AB. The PF127 percentage (B) and the biocellulose percentage (A) were the most significant model terms in predicting G′ of the temperature-responsive hydrogel.

Furthermore, the developed quadratic model showed a high R^2^ with a value of 0.9816 and a high value of R^2^_adj_ (0.9684). The predicted R^2^ of 0.7992 was close to R^2^_adj_. The value of adequate precision was greater than 4 (24.0200 as referred to Table 3), signifying that an adequate signal is considered for the model in predicting G′ within the range of study [28]. Equation (2) describes the developed quadratic model for G′ in terms of actual factors after excluding the non-statistically significant model terms.

(2) $$\ln(Y_{2})=+2.93+0.9538A+3.56B-0.5377A^{2}-2.24B^{2}$$

subjected to: 0 *w*/*v*% ≤ A ≤ 3.62 *w*/*v*%, and 10.86 *w*/*v*% ≤ B ≤ 39.14 *w*/*v*%

**Figure 4:**
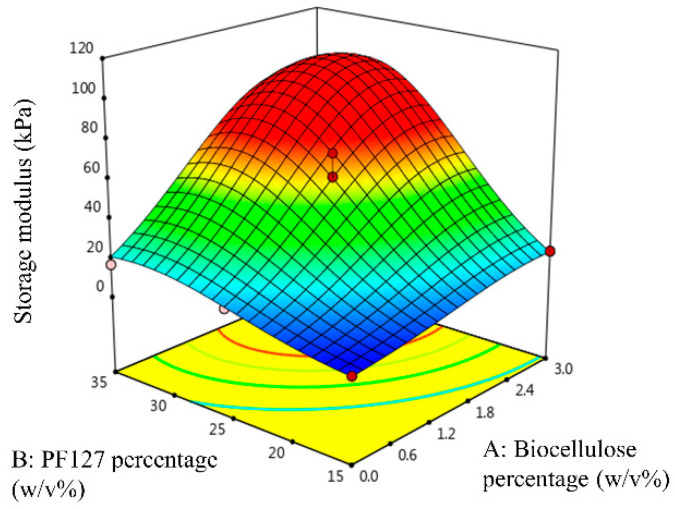
Three-dimensional response surface of G′ as a function of biocellulose percentage and PF127 percentage.

Figure 4 shows the three-dimensional response surface of G′ as a function of the biocellulose percentage and the PF127 percentage. As shown in Figure 4, high G′ was attained at a high biocellulose percentage and a high PF127 percentage. The G′ of the temperature-responsive hydrogels was increased with increase in PF127. Additional PF127 micelles contained in the temperature-responsive hydrogel matrix will increase aggregation of unimers and result in the formation of a stronger solid-like hydrogel with higher G′ [22]. On the other hand, the G′ of the temperature-responsive hydrogel was also increased with increase in the biocellulose percentage in the hydrogel formulation. This is because biocellulose binds with hydrophilic poly(ethylene oxide) chains through intermolecular hydrogen bonding [23]. This bonding increases the strength of temperature-responsive hydrogels and their cross-linking density, therefore increasing the G′ of the temperature-responsive hydrogel.

#### 2.2.2. Statistical Model and Analysis of t_50%_

The ANOVA results for the quadratic model for t_50%_ are shown in Table 4. Similar to LCST and G′, the Prob > F value of the quadratic model was less than 0.05 (<0.0001 according to Table 4). Additionally, the Prob > F value for lack of fit was 0.7336 (>0.05). Based on the Prob > F value, the model terms of the developed quadratic model were arranged in significance ranking as follows: B > A > B^2^ > AB > A^2^. Unsurprisingly, the PF127 percentage (B) was the most significant model term in predicting t_50%_ of the temperature-responsive hydrogel. This can be explained by the fact that the PF127 percentage was the dominant component in the temperature-responsive hydrogel formula.

The developed quadratic model also showed high R^2^ (0.9664) and R^2^_adj_ (0.9423), as shown in Table 4. According to Table 4, the predicted R^2^ of 0.8660 was close to the R^2^_adj_, which had a value of 0.9423. In addition, the value of adequate precision was 19.8750. Equation (3) shows the quadratic model for t_50%_ in terms of actual factors after excluding the non-statistically significant model terms.

(3) $$Y_{3}=+9.68+4.83A+18.86B-6.94B^{2}$$

subjected to: 0 *w*/*v*% ≤ A ≤ 3.62 *w*/*v*%, and 10.86 *w*/*v*% ≤ B ≤ 39.14 *w*/*v*%

**Figure 5:**
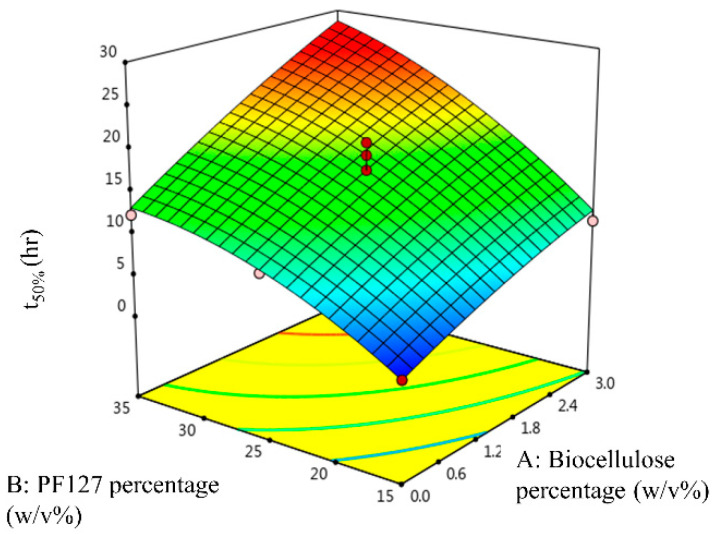
Three-dimensional response surface of t_50%_ as a function of biocellulose percentage and PF127 percentage.

Figure 5 shows the three-dimensional response surface of t_50%_ as a function of the biocellulose percentage and the PF127 percentage. As shown in Figure 5, high t_50%_ was attained at a high biocellulose percentage and a high PF127 percentage. t_50%_ of the temperature-responsive hydrogels was increased with increase in the PF127 percentage. Additional PF127 micelles contained in the temperature-responsive hydrogel at higher PF127 percentage drive the aggregation of unimers and lead to strong interlocking of SSD within the temperature-responsive hydrogel network, prolonging the sustained released of SSD with higher t_50%_. On the other hand, the t_50%_ of the temperature-responsive hydrogel was also increased with increase in the biocellulose percentage in the hydrogel formulation. This postulation was confirmed by the three-dimensional response surface of t_50%_ depicted in Figure 5. This occurs because biocellulose interacts with PF127 and leads to strong interlocking of SSD within the temperature-responsive hydrogel network, prolonging the sustained released of SSD.

#### 2.2.3. Statistical Model and Analysis of Inhibition Zone Diameter against *S. aureus*

The ANOVA results for the quadratic model for inhibition zone diameter against *S. aureus* are shown in Table 5. Similar to the previously discussed responses, the Prob > F value of the quadratic model had a value of 0.0015 (refer to Table 5), which was smaller than 0.05. Likewise, the Prob > F value for lack of fit was 0.7989 (>0.05). In addition, the quadratic model consisted of five model terms, namely, A, B, AB, A^2^, and B^2^. The significance ranking was arranged in accordance with B > A > A^2^ > B^2^ > AB, based on the Prob > F value. The most significant model term in predicting the inhibition zone diameter against *S. aureus* of the temperature-responsive hydrogel was the PF127 percentage (B) due to its dominant representation in the temperature-responsive hydrogel formula.

Further, the developed quadratic model showed high R^2^ (0.9097) and R^2^_adj_ (0.8452), as shown in Table 5. In addition, the predicted R^2^ of 0.6665 was close to the R^2^_adj_. The value of adequate precision was 13.4169, as shown in Table 5. Equation (4) describes the quadratic model for the inhibition zone diameter against *S. aureus* in terms of actual factors after excluding the non-statistically significant model terms.

(4) $$Y_{4}=+18.93+1.91A+4.98B+1.24A^{2}-2.64B^{2}$$

subjected to: 0 *w*/*v*% ≤ A ≤ 3.62 *w*/*v*%, and 10.86 *w*/*v*% ≤ B ≤ 39.14 *w*/*v*%

**Figure 6:**
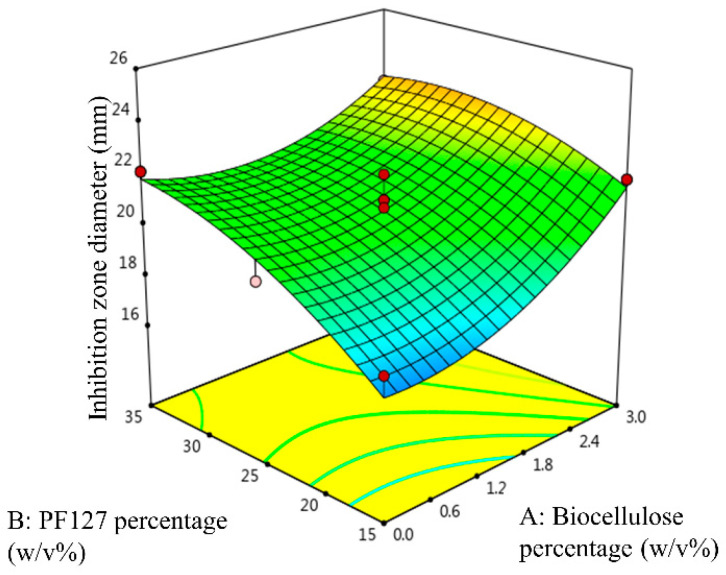
Three-dimensional response surface of inhibition zone diameter as a function of biocellulose percentage and PF127 percentage.

Figure 6 shows the three-dimensional response surface of the inhibition zone diameter against *S. aureus* as a function of the biocellulose percentage and the PF127 percentage. As shown in Figure 6, a high inhibition zone diameter against *S. aureus* was attained at a high biocellulose percentage and a high PF127 percentage. The inhibition zone diameter against *S. aureus* increased with increase in the PF127 percentage in the temperature-responsive hydrogel. On the other hand, the inhibition zone diameter against *S. aureus* was also increased with increase in the biocellulose percentage in the temperature-responsive hydrogel. A confirmation of this postulation is provided by the three-dimensional response surface of the inhibition zone diameter against *S. aureus* depicted in Figure 6. This can be explained by sustained release of the SSD drug from the temperature-responsive hydrogel at high biocellulose content. Sustained SSD drug release improves the inhibitory potential of the drug against bacterial strains by maintaining constant and localized release of the drug [24,25].

#### 2.2.4. Verification of Regression Model on Diagnostic Plot

The adequacy of quadratic models for LCST, G′, t_50%_, and the inhibition zone diameter against *S. aureus* was verified through different types of diagnostic plots, which were: normal % probability vs. internally studentised residual plot, predicted vs. actual values plot, and finally internally studentised residuals vs. number of runs plot, as shown in Figure S1, Figure S2, and Figure S3, respectively in the Supplementary Materials. As presented in Figure S1, no abnormal experimental results were shown in the plot for LCST, G′, t_50%_, and the inhibition zone diameter models. The majority of the residuals were distributed normally and located close to the straight line. At the same time, a good correlation was observed between the actual values and the predicted values with minor scattering, as demonstrated in Figure S2. This trend confirmed the adequacy of the quadratic models in predicting the response for LCST, G′, t_50%_, and the inhibition zone diameter. Figure S3 shows the random distribution of residuals in between the interval of ±3.00, suggesting a good approximation of the models with the constant variance assumption [29,30].

#### 2.2.5. Optimisation and Model Validation

The biocellulose percentage (A) and the PF127 percentage (B) were optimised to achieve LCST between 28–32 °C (near to the body surface temperature) and maximum values of G′, t_50%_, and the inhibition zone diameter. The optimised biocellulose percentage and PF127 percentage were 3.000 *w*/*v*% and 19.047 *w*/*v*%, respectively. These optimised process variables were expected to have LCST at 28.000 °C, G′ of 37.455 kPa, t_50%_ of 15.659 h, and an inhibition zone diameter of 22.388 mm, with desirability of 0.68 as shown in Table 6.

**Figure 7:**
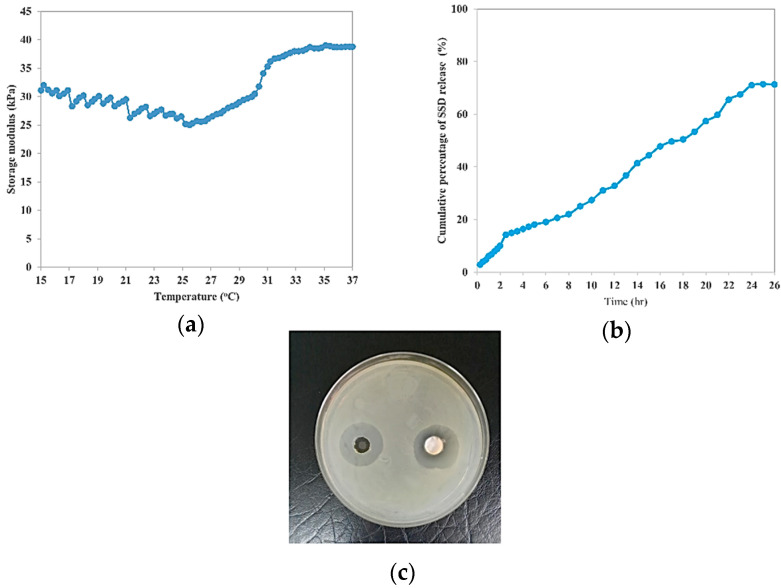
(**a**) LCST and storage modulus (G′) of optimum temperature-responsive hydrogel formulation as a function of temperature, (**b**) cumulative percentage of SSD release from optimum temperature-responsive hydrogel formulation, and (**c**) photographs of inhibition zone on optimum SSD-loaded temperature-responsive hydrogel formulation against *S. aureus*.

To confirm the optimum process variables that were predicted by CCD, experimental runs with the suggested optimum process variables were conducted. The responses are plotted in Figure 7a–c for LCST and G′, t_50%_, and the inhibition zone diameter, respectively. Table 7 summarises the experimental values of the responses and the percentage of error for the optimised temperature-responsive hydrogel formula at 3.000 *w*/*v*% biocellulose percentage and 19.047 *w*/*v*% PF127 percentage. The experimental values of the responses (LCST, G′, t_50%_, and inhibition zone diameter) were in good agreement to the predicted responses where the calculated absolute value of the percentage of error for all responses was less than 10.00%. This indicates that the developed quadratic models showed reasonably satisfactory optimisation to produce the desired LCST, G′, t_50%_, and inhibition zone diameter by varying the process variables.

### 2.3. In Vitro Cytotoxicity Test

**Figure 8:**
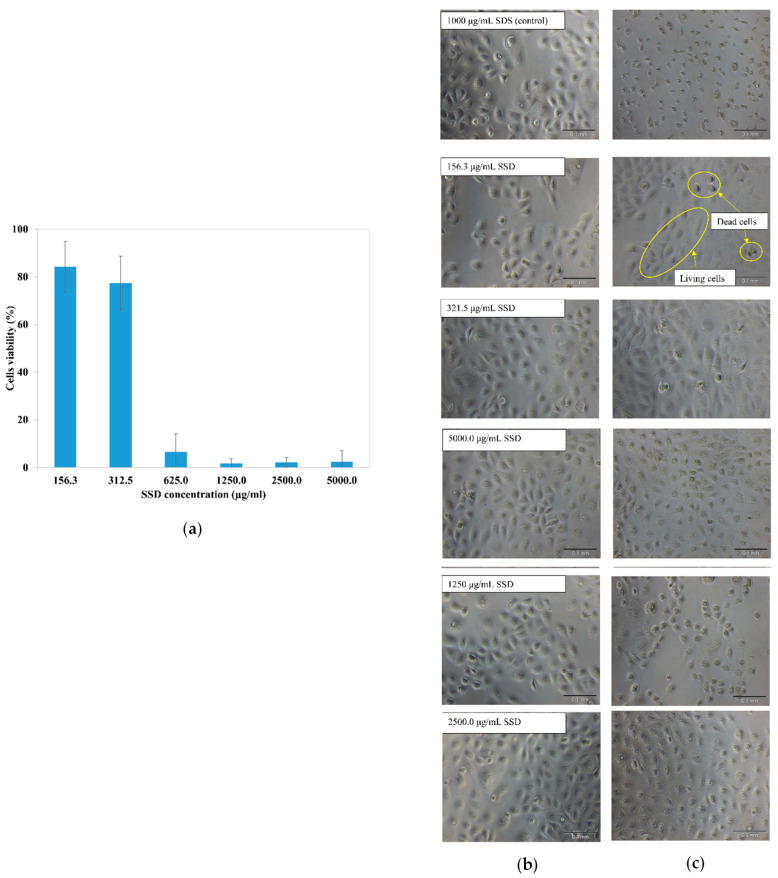
(**a**) HaCaT cell viability after treatment with temperature-responsive hydrogel loaded at different SSD concentrations, (**b**) HaCaT cell morphology before treatment, and (**c**) after 24 h of treatment with SSD-loaded temperature-responsive hydrogel at indicated concentrations.

Figure 8a shows the HaCaT cell viability after treatment with temperature-responsive hydrogel loaded at different SSD concentrations. As represented by Figure 8a, the viability of HaCaT cells decreased with increasing SSD drug concentration. This was probably due to cytotoxicity of the SSD drug against the HaCaT cells at high concentration [31,32]. According to the ISO 10993-5 standard, when the cell viability is more than 70%, this means no cytotoxic potential of the testing material. As presented in Figure 8a, the cell viability at 312.5 μg/mL SSD was recorded as 77.34 ± 11.3% (cell death 22.66%), whereas the cell viability drastically dropped at higher SSD concentration. This indicates that the SSD-loaded temperature-responsive hydrogel formulation with SSD concentration of 312.5 μg/mL and less could be classified as non-cytotoxic. Commercial SSD cream consists of 1 *w*/*w*% SSD drug. According to pharmacokinetics study and absorption of the commercial 1 *w*/*w*% SSD cream, the serum sulfadiazine concentration is proportional to the amount of cream applied, as well as to the extent of the burned area. The maximum serum sulfadiazine level was recorded at 80 to 120 μg/mL [33]. This was supported by a study conducted by MI et al. (1985) which found that the maximum sulfadiazine concentration of a severely burned patient receiving 1 *w*/*w*% commercial SSD cream was 91 μg/mL after 24 h of treatment [34]. The maximum sulfadiazine concentration in body serum (80 to 120 μg/mL) was less than the cytotoxic limit of SSD-temperature-responsive hydrogel (>312.5 μg/mL) on HaCaT cells. This confirms the safe use of the SSD-loaded temperature-responsive hydrogel on human skin.

Figure 8b shows the morphologies of the HaCaT cells before treatment and after 24 h of treatment with SSD-loaded temperature-responsive hydrogel at the indicated concentration. As demonstrated in Figure 8b, most of the HaCaT cells that were exposed to 156.3 and 312.5 μg/mL SSD were of spindle shape with tight packing between cells, similar to the living cells before treatment with the SSD-loaded temperature-responsive hydrogel. However, the number of living cells was dramatically decreased and dead HaCaT cells with cuboidal shape and loosely packed were observed at concentrations higher than 312.5 μg/mL SSD. In summary, the SSD-loaded temperature-responsive hydrogel can be used as a safe replacement for commercial SSD cream at application concentrations with no toxic effect.

### 2.4. In Vivo (Animal) Dermal Test

#### 2.4.1. Dermal Sensitization

Table 8 summarizes the number of animals that showed results for the response indices at 24 and 48 h followed by patch removal during the challenge phase. Tables S1–S3 in the Supplementary Materials present the test animal sequence, animal number, sex, initial body weight, and body weight after challenge for the test animal, negative, and positive groups. Similar to the negative controls, the test animals exhibited no results for the response indices (both erythema and edema) on guinea pig skin at 24 and 48 h followed by patch removal during the challenge phase (Table 8). However, the tested positive control animals revealed skin erythema and edema at 24 and 48 h followed by patch removal during the challenge phase.

The intensity of skin reactions of guinea pigs was scored in accordance with the Magnusson and Kligman grading [35]. The test animals and negative controls scored 0, that is, no visible change occurred on the skin of guinea pigs at 24 and 48 h followed by patch removal. In contrast, the positive control animals scored 2 and 3, indicating moderate to intense skin reaction and confluent erythema on the skin of the guinea pigs. The results of the dermal sensitization test revealed that the SSD-loaded temperature-responsive hydrogel did not produce a skin sensitization effect on the guinea pigs.

#### 2.4.2. Animal Irritation

Tables S4–S6 in the Supplementary Materials summarise the SRSs of rabbits at 1, 24, 48, and 72 h after the application of SSD-temperature-responsive hydrogel and for the negative and positive control tests. Table 9 and Table 10 show the PIS and PII of the SSD-loaded temperature-responsive hydrogel, negative control, and positive control groups during the observation period. All three rabbits treated with SSD-loaded temperature-responsive hydrogel and negative control appeared active and healthy, with no sign of adverse pharmacological effects, gross toxicity, nor abnormal behavior. In addition, no irreversible alterations were observed on the skin of animals treated with the SSD-loaded temperature-responsive hydrogel and negative control treatment throughout the 72 h observation period (Tables S4 and S5 in Supplementary Materials). As shown in Table 9, the PIS and PII of the SSD-loaded temperature-responsive hydrogel and negative control were 0, which indicated that no erythema and/nor edema was observed on rabbit skin after the application of SSD-loaded temperature-responsive hydrogel and negative control treatment. Comparatively, the positive control animals had SRSs of 3 and 4 (Table S6 in Supplementary Materials). Moderate to severe erythema and edema were observed on the rabbit skin after the positive control treatment. The PIS and PII of the positive controls were both 8, which is the maximum possible score for irritation. In conclusion, the SSD-loaded temperature-responsive hydrogel did not cause any irritant response on rabbit skin.

## 3. Conclusions

Optimisation of temperature-responsive hydrogel formulation as a drug delivery system was conducted using RSM via the CCD method. The developed quadratic models, which set the responses of LCST near to the body surface temperature, maximum value of G′, t_50%_, and maximum inhibition zone diameter, suggested that the optimum temperature-responsive hydrogel formulation was 3.000 *w*/*v*% biocellulose percentage and 19.047 *w*/*v*% PF127 percentage. Correspondingly, the experimental values of the LCST, G′, t_50%_, and inhibition zone diameter under the optimum process variables were determined as 28.000 °C, 37.455 kPa, 15.659 h, and 22.388 mm, respectively, with error percentages of −7.692%, 3.479%, 9.327%, and 4.181%, respectively. This was found to be in good agreement with the predicted optimised LCST, G′, t_50%_, and inhibition zone diameter values according to the mathematical model. This confirmed the validity of the mathematical model developed by RSM in this study to approximate the characterisation and performance of the temperature-responsive hydrogel during drug delivery. Moreover, in vitro cytotoxicity testing against HaCaT cells was performed to evaluate the toxicity of the optimum temperature-responsive hydrogel suggested by RSM. It was found that the SSD-loaded temperature-responsive hydrogel could be a safe replacement for the commercial SSD cream with no toxic effect on the HaCaT cells. Last, but not least, in vivo (animal) dermal testing, including both dermal sensitization and animal irritation, was conducted to evaluate the safety and biocompatibility of the optimised formula. The results of the dermal sensitization revealed that no sensitization effects on the skin of guinea pigs at either 24 h or 48 h following patch removal occurred. In addition, the results of animal irritation testing showed no irreversible alterations on the skin treated with the SSD-loaded temperature-responsive hydrogel throughout the 72 h observation period. The SSD-loaded temperature-responsive hydrogel did not cause an irritant response.

## 4. Materials and Methods

### 4.1. Materials

Oil palm empty fruit bunches (OPEFBs) were gathered from a palm oil mill namely Tennamaram, Selangor, Malaysia. Pluronic F127 (PF127) (molecular weight (MW): 12,600 g/mol), dimethylsulfoxide (DMSO), 3-(4,5-dimethylthiazol-2-yl)-2,5-diphenyltetrazolium bromide (MTT), 1-chloro-2,4-dinitrobenzene (DNCB), and sodium lauryl sulfate (SLS) were supplied by Sigma–Aldrich, Hamburg, Germany. SSD was supplied by the Tokyo Chemical Industry, Tokyo, Japan. Human epidermal keratinocyte (HaCaT) cells were purchased from the American Type Culture Collection (ATCC), Virginia, USA. Fetal bovine serum (FBS), penicillin/streptomycin antibiotic, and Dulbecco′s modified Eagle′s medium high glucose (DMEM-HG) were supplied from Thermo Fisher Scientific, Waltham, MA, USA. Ammonia solution (25 *v*/*v*%) was supplied by Guangdong Guanghua Sci-Tech Co. Ltd., Shantou, China. Staphylococcus aureus (*S. aureus*) (ATCC^®^ 6538) was purchased from Microbiologics Inc., Saint Cloud, MN, USA. Nutrient broth and nutrient agar were obtained from HiMedia Laboratories Pvt. Ltd., Ambernath, India. Normal saline was supplied by B. Braun Medical Inc., Melsungen, Germany. Ethanol was purchased from Systerm chemicals, Shah Alam, Malaysia. Finally, sodium dodecyl sulphate (SDS) was provided by the Promega Corporation, Madison, WI, USA. All chemicals were American Chemical Society (ACS) grade and were used as received.

#### 4.1.1. Synthesis of Temperature-Responsive Hydrogel

OPEFBs were the source of biocellulose; the extraction process was performed following the methodology described in our previous study [36]. Additionally, the temperature-responsive hydrogels were synthesized via a cold method [20]. Typically, different weight/volume percentage of PF127 was dissolved in deionized water and kept in a refrigerator at 2–8 °C for 20 h until complete dissolution. Following this, pre-determined extracted biocellulose was added into the PF127 solution. The combined solution was then stirred for 7 days at 200 rpm at a temperature between 2–8 °C in order to obtain a homogeneous temperature-responsive hydrogel.

#### 4.1.2. Optimisation of Temperature-Responsive Hydrogel Formulation

RSM was used to determine the optimum formulation of the temperature-responsive hydrogel for its application as a topical drug delivery system. Generally, optimisation by RSM involves three major steps, namely, design of experiments (DOE), model fitting and statistical analysis, and condition optimisation.

#### 4.1.3. DOE

The central composite design (CCD) technique was used with the aid of Design Expert software version 7.0.0 (Stat-Ease Inc., Minneapolis, MN, USA). CCD is a standard, effective, and the most used, design of RSM. It is ideal for estimating the main effects of variables and the interactions between them through a rationalised number of experimental runs, along with the ability to develop a higher polynomial response model with a smaller number of factors [26].

In this study, two independent process variables, namely, the biocellulose *w*/*v*% percentage (A) and the PF127 *w*/*v*% percentage (B), were selected as the studied effects. The range of each independent process variables was selected based on our previous study [20]. An amount of 15–35 *w*/*v*% PF127 was selected due to the temperature-responsive properties of PF127 within this range [37,38,39]. On the other hand, 0–3 *w*/*v*% biocellulose was selected because a homogenous hydrogel solution was not able to be produced once the biocellulose concentration exceeded 3 *w*/*v*%. In this study, 5 levels were used including high level (+1), low level (−1), and centre point (0), in addition to 2 outer points corresponding to (-𝛼) and (+𝛼). Alpha (𝛼) has a maximum value of (2n/4);, it is well-defined as a distance from the centre point, where (n) is the number of independent process variables [40]. Accordingly, the value of α in this study was 1.41421. In addition, DOE in this study included 6 centre points to reduce the experimental error. There were 14 experimental runs in total. Table 11 shows the ranges of the independent process variables. The DOE variables for the optimisation of temperature-responsive hydrogel formulation based on RSM are shown in Table 12.

#### 4.1.4. Model Fitting and Statistical Analysis

The predictive model for each response was developed as part of the RSM application. Four responses, namely, lower critical solution temperature (LCST), G′, half-life of SSD release (t_50%_), and inhibition zone diameter against *S. aureus*, were selected as the desired responses. *S. aureus* was selected as it is the major cause of morbidity and death in burns [41]. Analysis of variance (ANOVA) was utilized to test the accuracy and significance of the developed models. Both coefficient R^2^ and the adjusted R^2^ values were used to determine the accuracy of the fitted model, while the model statistical significance was evaluated using the 𝐹-value. The probability value (*p*-value) was used to evaluate the significance of the model at the 95% confidence level.

#### 4.1.5. Condition Optimisation

After validation of the developed models, a three-dimensional contour plot was obtained (known as a three-dimensional response surface) according to the mathematical analysis of the experimental data in order to visualise the interaction between different independent process variables and their impact on the four responses. The optimum process variables with LCST ranged between 28–32 °C near to the body surface temperature [42]; the highest value of G′ and t_50%_, and the highest inhibition zone diameter, were identified.

After obtaining the optimum independent process variables using RSM, a confirmatory run of the experiment was performed and evaluated against the predicted response from the model. Equation (5) was used to calculate the percentage of error between the experimental and the predicted values.

(5) $$\begin{array}{cc} Percentage\;\;of & error\;\left(\%\right)\\ & =\frac{Experimental\;value-Predicted\;value}{Experimental\;value}\times 100\;\%\; \end{array}$$

#### 4.1.6. In Vitro Cytotoxicity Test

An MTT assay method was followed to find out the in vitro cytotoxicity of the optimum temperature-responsive hydrogel against HaCaT cells. In general, the MTT assay method includes two main steps, including, HaCaT cell culture and MTT assay, which are described in the following sections.

#### 4.1.7. HaCaT Cell Culture

HaCaT cells were seeded and grown in DMEM-HG with 10 *v*/*v*% FBS and 1 *w*/*v*% streptomycin/penicillin antibiotic [43]. Then, HaCaT cells were incubated under 5 *v*/*v*% carbon dioxide (CO_2_) supplied at 37 °C for 20 h. Then, HaCaT cells were plated until reaching 70% confluency. Finally, the temperature-responsive hydrogel was dissolved and diluted in DMEM-HG with different concentrations ranging from 156.3 μg/mL to 5000.0 μg/mL.

#### 4.1.8. MTT Assay

The International Organization for Standardization (ISO) standard number:10993-5 was followed to perform the MTT assay [44]. Initially, the cells of HaCaT were seeded into a 96-well plate with a density of 3000 HaCaT cells/well. Temperature-responsive hydrogel was added to each well with a 100 µL final volume. The HaCaT cells were then incubated for 24 h. Later, 5 mg/mL MTT with a volume of 10 µL was loaded into each well; therefore, the final concentration was 0.45 mg/mL. Next, the plate was incubated for 4 h. Afterwards, 100 µL of DMSO was added into the HaCaT cells and the absorbance was measured at a wavelength of 570 nm. SDS was used as a positive control, whereas the negative control was the cells before treatment with temperature-responsive hydrogel or SDS. The percentage cell viability was calculated using Equation (6).

(6) $$Cell\;viability\;\left(\%\right)=\frac{Absorbance\;of\;sample}{Absorbance\;of\;blank}\times 100\%$$

The HaCaT cell morphologies before treatment and after 24 h of SSD-loaded temperature-responsive hydrogel treatment were observed using microscope ckx41 (Olympus, Tokyo, Japan) under 100× magnification.

#### 4.1.9. In Vivo (Animal) Dermal Test

Dermal sensitization and animal irritation tests were performed in accordance with the ISO10993-10 standards [35]. All procedures involving the use and care of animals adhered to the Universiti Kebangsaan Malaysia Research and Ethics Committee approval code: BIOSERASI/UKM/2021/MIMI NORHILDA/30- JUNE/1188-JUNE-2021-JUNE-2022.

#### 4.1.10. Dermal Sensitization

A closed-patch test (Buehler test) was conducted to assess the results of the dermal sensitization assay of the temperature-responsive hydrogel. The Buehler test consists of two major phases, namely, the induction and challenge phases, as described in the following sub-sections. Healthy albino Dunkin–Hartley guinea pigs were assigned to two groups. Ten guinea pigs were used as the test group and received temperature-responsive hydrogel formulation, whereas five guinea pigs were used as a negative control group and received normal saline. Ten guinea pigs were used as a positive control group and received 0.08 *v*/*v*% 1-chloro-2,4-dinitrobenzene and 80 *v*/*v*% ethanol). A 10 cm × 15 cm fur area was shaved on the guinea pigs’ backs prior to the test.

#### 4.1.11. Induction Phase

**Figure 9:**
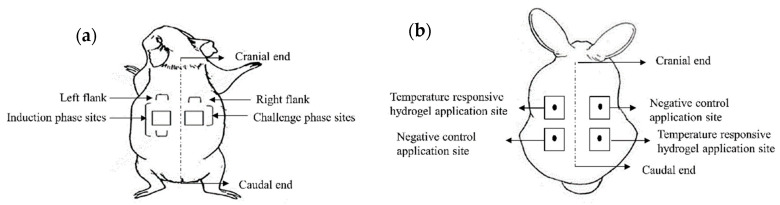
Arrangement of application sites for (**a**) dermal sensitization assay, and (**b**) animal irritation test.

An 8 cm^2^ patch was first soaked in the temperature-responsive hydrogel and applied onto the upper left flank on the back of each guinea pig to cover the induction phase sites (Figure 9a). The patch was secured with an occlusive dressing. On the other hand, negative controls were treated with normal saline. The patches were removed after 6 h. The test was repeated three days a week for three weeks.

#### 4.1.12. Challenge Phase

The guinea pigs from the test group were treated with the temperature-responsive hydrogel after 14 days of the induction phase. The same procedures were applied in the challenge phase; that is, patches were applied at the upper right flank on the back of each guinea pig to cover the challenge phase sites (Figure 9a). The patches were removed after 6 h.

The appearance of each application site was observed at 24 and 48 h after patch application. Full-spectrum lighting was used to visualize skin reactions. Magnusson and Kligman grading was used to describe and grade skin reactions for erythema and edema on each challenge site at different intervals [35].

#### 4.1.13. Animal Irritation

An animal irritation test was carried out on three New Zealand white rabbits. A 10 cm × 15 cm fur area was shaved on each New Zealand white rabbits′ back prior to the test. A 2.5 cm × 2.5 cm patch was first soaked in the temperature-responsive hydrogel, applied onto two separate sites on the back of each rabbit (Figure 9b), and secured with an occlusive dressing. The negative controls were treated with normal saline. The positive controls received 20 *w*/*v*% sodium lauryl sulfate in deionized water. The patches were removed after 4 h.

The appearance of each application site was observed at 1, 24, 48, and 72 h after the application. Full-spectrum lighting was used to visualize the skin reactions, which were described and graded for erythema and edema following the scoring system shown in Table 13 [35]. The skin reaction score (SRS) of each application site at different intervals was calculated using Equation (7). The primary irritation score (PIS) of the test and control animals and the primary irritation index (PII) of the temperature-responsive hydrogel were calculated using Equations (8) and (9), respectively.

(7) $$SRS={\left(SRS\right)}_{erythema}+{\left(SRS\right)}_{oedema}$$

(8) $$PIS=\frac{{\left(SRS\right)}_{24h}+{\left(SRS\right)}_{48h}+{\left(SRS\right)}_{72h}}{Number\;of\;test\;sites\times Number\;of\;time\;points}$$

(9) $$\begin{array}{c} {\left(PII\right)}_{Thermo-responsive\;cellulose\;hydrogel}\\ =\frac{{\left(PIS\right)}_{test\;animals}-\;{\left(PIS\right)}_{control\;animals}\;}{Number\;of\;test\;animals}\; \end{array}$$

### 4.2. Characterisation of Temperature-Responsive Hydrogel

#### Rheological Property

A Physica MCR301 rheometer (Anton Paar, Graz, Austria) was used to measure the rheological properties of the synthesised temperature-responsive hydrogel. The heating rate was 1 °C/min with a temperature range between 15 °C (non-physiological condition) and 37 °C (physiological condition). The storage modulus (G′) value was recorded at a fixed angular frequency (10 1/s) as a function of temperature. A controlled shear stress (Css) mode was used during this test with a Css constant of 245 Pa/mN.m and amplitude gamma = 0.5%.

### 4.3. Performance Assessment of Temperature-Responsive Hydrogel

#### 4.3.1. In Vitro Drug Delivery Study

A quantity of 100 mg of SSD was loaded into 10 g of temperature-responsive hydrogel at sol-phase. Next, the SSD-loaded temperature-responsive hydrogel was stirred at 200 rpm for 1 h under low temperature (2–8 °C) to obtain a homogeneous drug-loaded temperature-responsive hydrogel [45]. A vertical diffusion cell, Copley HDT 1000 (COPLEY, UK) was used for a temperature-responsive hydrogel in vitro drug delivery study. The methodology used to perform the in vitro drug delivery study followed the methodology described in our previous work [20,46]. The concentration of SSD in the receptor medium was analyzed using a UV-visible spectrophotometer, Genesys 10S UV-VIS (Thermo scientific, Waltham, MA, USA), at a wavelength of 260 nm. Equation (10) was used to calculate the cumulative percentage of SSD released from the temperature-responsive hydrogel [47].

(10) $$\begin{array}{cc} Cumulative\;\;percentage & of\;\;SSD\;\;release\;\;\left(\%\right)\\ & =\left[\frac{C_{SSD\left(t\right)}\;V_{rm}+v\sum_{1}^{t-1}C_{SSD\left(t\right)}}{W_{SSD}}\right]\times 100\;\% \end{array}$$

where *C_SSD_*_(*t*)_ is the concentration of SSD drug released at time (*t*) (mg/mL), *v* is the volume of the withdrawn receptor medium (mL), *V_rm_* is the volume of the receptor medium (mL), and *W_SSD_* is the SSD drug amount loaded initially in the temperature-responsive hydrogel (mg).

#### 4.3.2. Antimicrobial Activity

The antimicrobial activity of the temperature-responsive hydrogel was indicated by the diameter of its zone of inhibition against *S. aureus*. Firstly, the nutrient agar medium was poured into a sterilized petri plate and allowed to solidify at room temperature. A quantity of 100 μL of broth bacterial suspension (10^8^ CFU/mL) was then spread on the nutrient agar surface using a sterile bent glass rod to prepare the confluent ground for *S. aureus* bacterial growth [16]. Subsequently, a 6 mm diameter well was created in the nutrient agar plate with a sterile tip. Next, 50 μL of drug-loaded temperature-responsive hydrogel was placed into the well. Lastly, the agar plate was incubated for 24 h at 37 °C. After 24 h of incubation, the diameter of the inhibition zone was measured using ImageJ software (NIH, Bethesda, MD, USA). A quantity of 1 *w*/*w*% of dissolved SSD drug in 0.25 *v*/*v*% ammonia phosphate buffer solution at pH 7.4 was used as the positive control.

## Figures and Tables

**Table 1:** CCD and experimental results of temperature-responsive hydrogel formulations.

Experimental Run	Independent Process Variables		Responses			
	Biocellulose, A (*w*/*v*%)	PF127, B (*w*/*v*%)	LCST, Y_1_ (°C)	G′, Y_2_ (kPa)	t_50%,_ Y_3_ (h)	Inhibition Zone Diameter, Y_4_ (mm)
1	1.50	25.00	19.00	55.00	16.00	21.00
2	0.00	15.00	45.30	2.70	1.75	18.10
3	1.50	25.00	19.00	59.00	16.50	22.00
4	3.00	15.00	38.40	17.70	9.22	21.80
5	1.50	25.00	18.00	50.00	15.00	20.00
6	3.00	35.00	15.00	61.40	26.43	23.00
7	0.00	35.00	15.80	16.80	12.24	22.10
8	1.50	10.86	51.30	3.00	1.98	16.00
9	3.62	25.00	17.50	73.90	25.56	25.00
10	1.50	25.00	19.50	54.00	18.28	19.00
11	1.50	25.00	18.00	71.00	14.00	20.70
12	0.00	25.00	21.30	13.90	9.32	19.60
13	1.50	39.14	11.70	68.50	24.12	21.10
14	1.50	25.00	20.00	59.00	19.80	21.00

**Table 2:** ANOVA of quadratic model for LCST.

Response	Model Terms	Sum of Squares (SS)	Degree of Freedom (DF)	Mean Square (MS)	F-Value	Prob > F	
Quadratic model		1889.65	5	377.93	215.08	<0.0001	Significant
	A	35.61	1	35.61	20.26	0.0028	
	B	1347.42	1	1347.42	766.81	<0.0001	
	AB	9.30	1	9.30	5.29	0.0549	
	A^2^	12.54	1	12.54	7.14	0.0319	
	B^2^	357.26	1	357.26	203.31	<0.0001	
Residual		12.30	7	1.76			
	Lack of Fit	9.47	3	3.16	4.46	0.0915	Not significant
	Pure Error	2.83	4	0.71			
Cor Total		1910.91	13				
R^2^ = 0.9935, R^2^_adj_ = 0.9889, Predicted R^2^ = 0.9611, Adequate precision = 41.0773							
A—biocellulose percentage, B—PF127 percentage							

**Table 3:** ANOVA of quadratic model for G′.

Response	Model Terms	SS	DF	MS	F-Value	Prob > F	
Quadratic model		16.41	5	3.28	74.58	<0.0001	Significant
	A	3.08	1	3.08	70.03	<0.0001	
	B	9.65	1	9.65	219.23	<0.0001	
	AB	0.0853	1	0.08	1.94	0.2063	
	A^2^	1.46	1	1.46	33.11	0.0007	
	B^2^	3.96	1	3.96	89.93	<0.0001	
Residual		0.3080	7	0.04			
	Lack of Fit	0.2552	3	0.08	6.44	0.0519	Not significant
	Pure Error	0.0528	4	0.01			
Cor Total		16.94	13				
R^2^ = 0.9816, R^2^_adj_ = 0.9684, Predicted R^2^ = 0.7992, Adequate precision = 24.0200							

**Table 4:** ANOVA of quadratic model for t_50%_.

Response	Model Terms	SS	DF	MS	F-value	Prob > F	
Quadratic model		737.95	5	147.59	40.21	<0.0001	Significant
	A	78.89	1	78.89	21.49	0.0024	
	B	271.13	1	271.13	73.86	<0.0001	
	AB	11.29	1	11.29	3.08	0.1229	
	A^2^	2.64	1	2.64	0.72	0.4243	
	B^2^	38.10	1	38.10	10.38	0.0146	
Residual		25.69	7	3.67			
	Lack of Fit	6.44	3	2.15	0.44	0.7336	Not significant
	Pure Error	19.26	4	4.81			
Cor Total		781.75	13				
R^2^ = 0.9664, R^2^_adj_ = 0.9423, Predicted R^2^ = 0.8660, Adequate precision = 19.8750							

**Table 5:** ANOVA of quadratic model for inhibition zone diameter against *S. aureus*.

Response	Model Terms	SS	DF	MS	F-Value	Prob > F	
Quadratic model		54.72	5	10.94	14.11	0.0015	Significant
	A	12.40	1	12.40	15.98	0.0052	
	B	18.92	1	18.92	24.39	0.0017	
	AB	1.96	1	1.96	2.53	0.1560	
	A^2^	7.69	1	7.69	9.91	0.0162	
	B^2^	5.53	1	5.53	7.13	0.0320	
Residual		5.43	7	0.78			
	Lack of Fit	1.10	3	0.37	0.34	0.7989	Not significant
	Pure Error	4.33	4	1.08			
Cor Total		62.39	13				
R^2^ = 0.9097, R^2^_adj_ = 0.8452, Predicted R^2^ = 0.6665, Adequate precision = 13.4169							

**Table 6:** Optimised process variables, predicted responses, and desirability.

Parameter		Unit	Value
Optimised process variables	Biocellulose percentage	*w*/*v*%	3.00
	PF127 percentage	*w*/*v*%	19.05
Predicted responses	LCST	°C	28.00
	G′	kPa	37.45
	t_50%_	hr	15.66
	Inhibition zone diameter	mm	22.39
Desirability	---	---	0.68

**Table 7:** Predicted and experimental values of responses and percentage of error.

Response	Predicted Value	Experimental Value	Percentage of Error (%)
LCST (°C)	28.00	26.00	−7.69
G′ (kPa)	37.45	38.80	3.48
t_50%_ (h)	15.66	17.26	9.33
Inhibition zone diameter (mm)	22.39	23.36	4.18

**Table 8:** Animals that showed results for the response indices at 24 and 48 h followed by patch removal during the challenge phase.

	Number of Animals Showed Response Index			
Response Index	Erythema	Oedema	Erythema	Oedema
Duration after patch removal	24 h		48 h	
Test animals (total number 10)	0 animals	0 animals	0 animals	0 animals
Negative control test animals (total number 5)	0 animals	0 animals	0 animals	0 animals
Positive control test animals (total number 10)	10 animals	10 animals	10 animals	10 animals

**Table 9:** PIS and PII of SSD-loaded temperature-responsive hydrogel and negative control during the observation period.

Animal Number	Animal Sex	PIS	
		SSD-Temperature-Responsive Hydrogel	Negative Control
r010L	Male	0	0
r012L	Male	0	0
r013L	Male	0	0
	PII	0	0

**Table 10:** PIS and PII of positive control during the observation period.

Animal Number	Animal Sex	PIS
r003L	Female	8
r004L	Male	8
r006L	Male	8
	PII	8

**Table 11:** The ranges of independent process variables.

Independent Process Variables		Coded Levels		
		−1	0	+1
A	*w*/*v*%	0.0	1.5	3.0
B	*w*/*v*%	15	25	35

**Table 12:** DOE variables for the optimisation of temperature-responsive hydrogel formulation based on RSM.

Experimental Run	Independent Process Variables	
	Biocellulose, A (*w*/*v*%)	PF127, B (*w*/*v*%)
1	1.50	25.00
2	0.00	15.00
3	1.50	25.00
4	3.00	15.00
5	1.50	25.00
6	3.00	35.00
7	0.00	35.00
8	1.50	10.86
9	3.62	25.00
10	1.50	25.00
11	1.50	25.00
12	0.00	25.00
13	1.50	39.14
14	1.50	25.00

**Table 13:** Scoring system of skin reaction for animal irritation test.

Reaction	Irritation Score
Erythema and eschar formation	
No erythema	0
Very slight erythema	1
Well-defined erythema	2
Moderate erythema	3
Severe erythema (beet-redness) to eschar formation preventing	4
Oedema formation	
No oedema	0
Very slight oedema	1
Well-defined oedema	2
Moderate oedema	3
Severe oedema	4
Maximal possible score for irritation	8

## Data Availability

Data is contained within the article.

## Supplementary Materials

The following supporting information can be downloaded at: https://www.mdpi.com/article/10.3390/gels9040329/s1, Figure S1: Plot of normal % probability versus internally studentised residual for (a) LCST (b) storage modulus (G′) (c) t50%, and (d) inhibition zone diameter; Figure S2: Plot of predicted versus actual values for (a) LCST (b) storage modulus (c) t50%, and (d) inhibition zone diameter; Figure S3: Plot of internally studentised residuals versus run number for (a) LCST (b) storage modulus (G′) (c) t50% and (d) inhibition zone diameter; Table S1: Information of test group animals during dermal sensitization test; Table S2: Information of positive control group animals during dermal sensitization test; Table S3: Information of negative control group animals during dermal sensitization test; Table S4: Skin reaction scores of test group animals during animal irritation test; Table S5: Skin reaction scores of negative control group animals during animal irritation test; Table S6: Skin reaction scores of positive control group animals during animal irritation test.
