# Supplementary material for: Temperature-Responsive Hydrogel for Silver Sulfadiazine Drug Delivery: Optimized Design and In Vitro/In Vivo Evaluation
Source: Gels. 2023 Apr 13;9(4):329. doi: 10.3390/gels9040329 (PMC10137830; doi:10.3390/gels9040329)
Supplement: Supplementary file 1 [file gels-09-00329-s001.zip › gels-2289863-supplementary.pdf]

Supplementary data

Verification of Regression Model on Diagnostic Plot

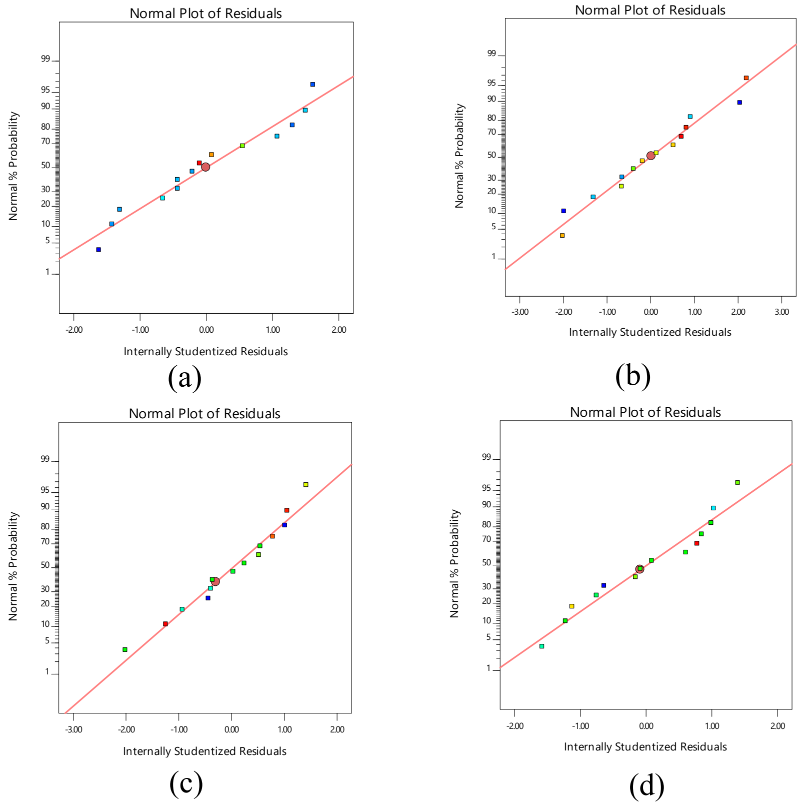

Figure S1. Plot of normal % probability versus internally studentised residual for (a) LCST (b) storage modulus (G') (c) $t_{50\%}$, and (d) inhibition zone diameter.

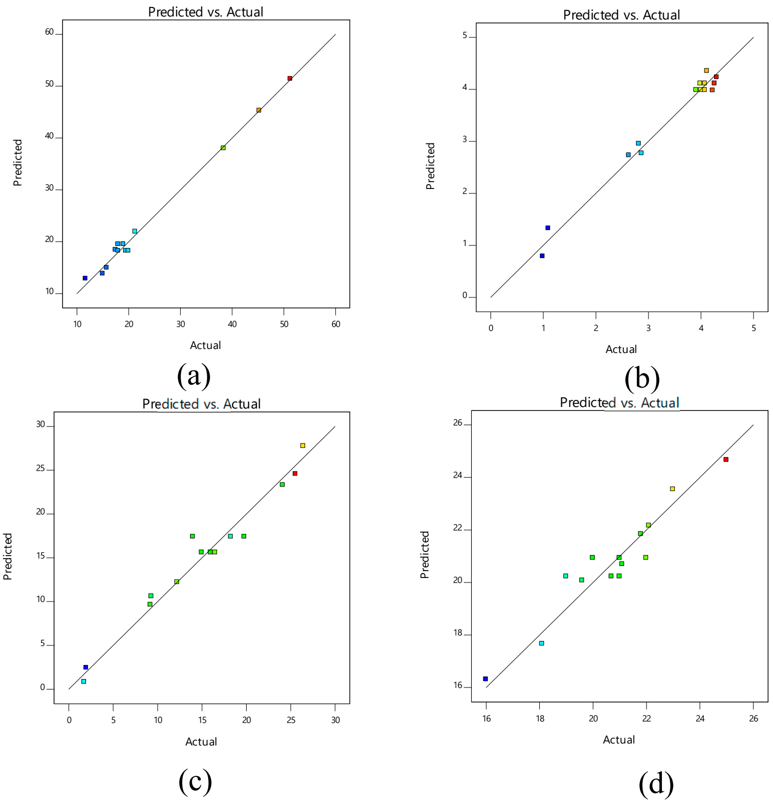

Figure S2. Plot of predicted versus actual values for (a) LCST (b) storage modulus (c) $t_{50\%}$, and (d) inhibition zone diameter.

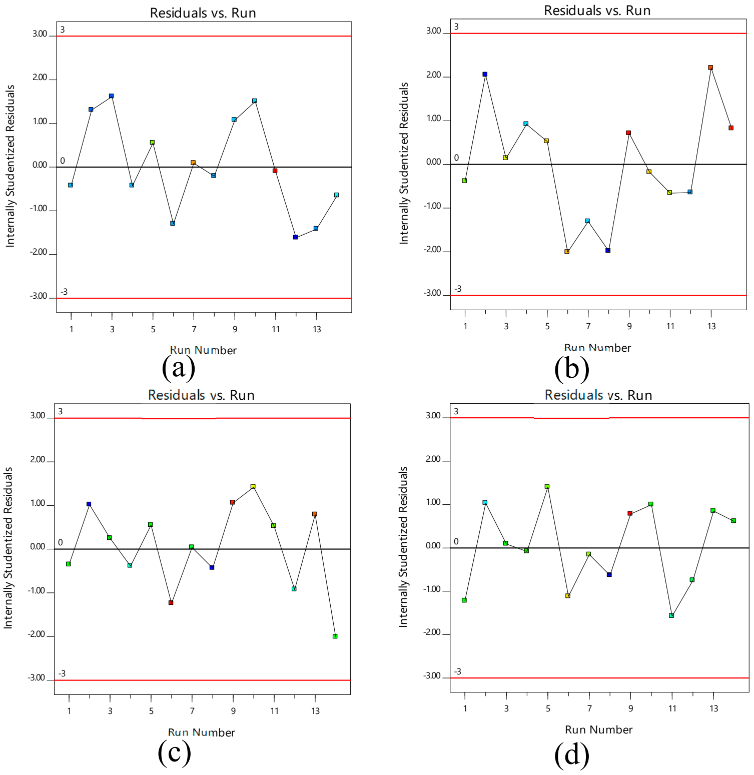

Figure S3. Plot of internally studentised residuals versus run number for (a) LCST (b) storage modulus (G') (c) $t_{50\%}$, and (d) inhibition zone diameter.

ANIMALS AINFORMATION AND SKIN REACTION SCORES DURING IN-VIVO DERMAL TEST

Information of animals during dermal sensitization test

Table S1 Information of test group animals during dermal sensitization test

Test animal sequence	Animal number	Sex	Initial body weight (g)	Body weight after challenge (g)
009-01	g176L	Male	378.18	533.35
009-02	g177L	Male	366.51	555.31
009-03	g178L	Male	361.79	493.97
009-04	g179L	Male	382.69	553.77
009-05	g180L	Male	446.66	544.58
009-06	g181L	Male	389.29	507.69
009-07	g182L	Male	377.53	526.23
009-08	g183L	Male	383.50	557.03
009-09	g184L	Male	393.41	502.68
009-10	g185L	Male	398.72	582.83

Table S2 Information of positive control group animals during dermal sensitization test

Test animal sequence	Animal number	Sex	Initial body weight (g)	Body weight after challenge (g)
PC-01	g011L	Male	461.90	660.08
PC-02	g012L	Male	470.16	619.73
PC-03	g013L	Male	433.08	421.10
PC-04	g014L	Male	3911.87	579.65
PC-05	g015L	Female	419.18	542.93
PC-06	g016L	Female	426.82	523.76
PC-07	g017L	Female	399.55	494.54

PC-08	g018L	Female	420.70	562.18
PC-09	g019L	Female	397.97	554.73
PC-10	g020L	Female	374.98	476.68

Table S3 Information of negative control group animals during dermal sensitization test

Test animal sequence	Animal number	Sex	Initial body weight (g)	Body weight after challenge (g)
NC-01	g171L	Female	377.64	512.59
NC-02	g172L	Female	356.45	621.88
NC-03	g173L	Male	422.61	573.10
NC-04	g174L	Male	488.53	577.85
NC-05	g175L	Male	414.54	546.28

Skin reactions scores during animal irritation test

Table S4 Skin reaction scores of test group animals during animal irritation test

Animal number	Sex	Test site	Score after 1 hour		Score after 24 hours		Score after 48 hours		Score after 72 hours	
			E	O	E	O	E	O	E	O
r010L	Male	Site 1	0	0	0	0	0	0	0	0
		Site 2	0	0	0	0	0	0	0	0
r012L	Male	Site 1	0	0	0	0	0	0	0	0
		Site 2	0	0	0	0	0	0	0	0
r013L	Male	Site 1	0	0	0	0	0	0	0	0
		Site 2	0	0	0	0	0	0	0	0

E: Erythema, O: Oedema

Table S5 Skin reaction scores of negative control group animals during animal irritation test

Animal number	Sex	Test site	Score after 1 hour		Score after 24 hours		Score after 48 hours		Score after 72 hours	
			E	O	E	O	E	O	E	O
r010L	Male	Site 1	0	0	0	0	0	0	0	0
		Site 2	0	0	0	0	0	0	0	0
r012L	Male	Site 1	0	0	0	0	0	0	0	0
		Site 2	0	0	0	0	0	0	0	0
r013L	Male	Site 1	0	0	0	0	0	0	0	0
		Site 2	0	0	0	0	0	0	0	0

Table S6 Skin reaction scores of positive control group animals during animal irritation test

Animal number	Sex	Test site	Score after 1 hour		Score after 24 hours		Score after 48 hours		Score after 72 hours	
			E	O	E	O	E	O	E	O
r003L	Female	Site 1	3	3	4	4	4	4	4	4
		Site 2	3	3	4	4	4	4	4	4
r004L	Male	Site 1	4	4	4	4	4	4	4	4
		Site 2	4	4	4	4	4	4	4	4
r006L	Male	Site 1	4	4	4	4	4	4	4	4
		Site 2	4	4	4	4	4	4	4	4
